# [*N*,*N*-Bis(2-pyridylmeth­yl)glycinato-κ^4^ *N*,*N*′,*N*′′,*O*]dichloridoiron(III)–[*N*,*N*-bis­(2-pyridylmeth­yl)glycine-κ^4^ *N*,*N*′,*N*′′,*O*]dichloridozinc(II) (1/1)

**DOI:** 10.1107/S1600536809051022

**Published:** 2009-11-28

**Authors:** Anne Nielsen, Christine J. McKenzie, Andrew D. Bond

**Affiliations:** a University of Southern Denmark, Department of Physics and Chemistry, Campusvej 55, 5230 Odense M, Denmark

## Abstract

The title compound, [Fe(C_14_H_14_N_3_O_2_)Cl_2_]·[ZnCl_2_(C_14_H_15_N_3_O_2_)], is formulated as [Fe^III^(bpg)Cl_2_][Zn^II^Cl_2_(bpgH)], where bpg is the tetra­dentate ligand *N*,*N*-bis­(2-pyridylmeth­yl)glycine. The structure contains one crystallographically distinct complex with Fe^III^ and Zn^II^ atoms present in a 50:50 ratio in a single-atom site. The non-coordinated O atoms of the carboxyl groups of bpg meet across crystallographic inversion centres, forming O—H⋯O hydrogen bonds that include only one H atom per two complexes, consistent with the 1:1 disorder of Fe^III^ and Zn^II^.

## Related literature

For related Fe^III^ structures of bpg, see: Mortensen *et al.* (2004 ▶). For details of the synthesis, see: Suzuki *et al.* (1988 ▶).
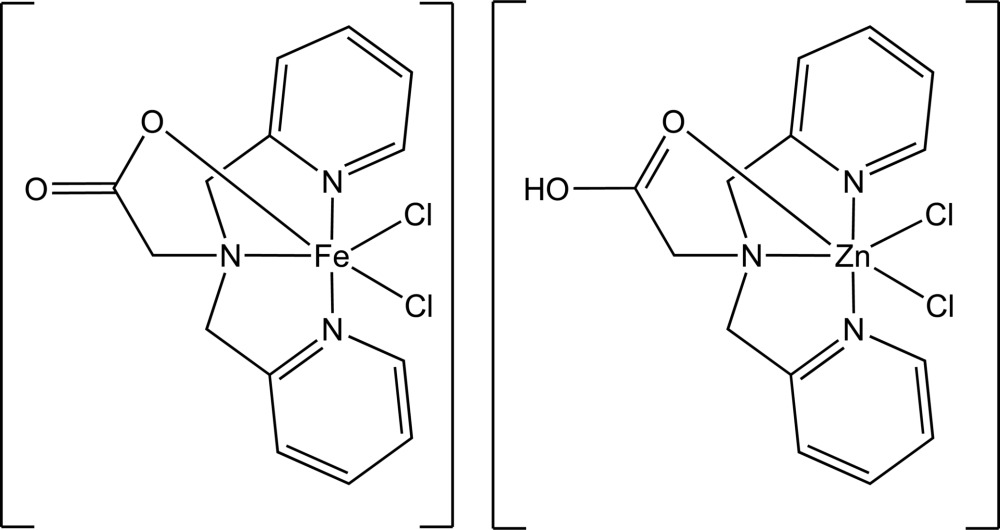


## Experimental

### 

#### Crystal data

[Fe(C_14_H_14_N_3_O_2_)Cl_2_]·[ZnCl_2_(C_14_H_15_N_3_O_2_)]
*M* *_r_* = 776.59
Monoclinic, 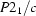

*a* = 8.8710 (3) Å
*b* = 13.1898 (5) Å
*c* = 13.3983 (4) Å
β = 91.737 (2)°
*V* = 1566.97 (9) Å^3^
*Z* = 2
Mo *K*α radiation
μ = 1.62 mm^−1^
*T* = 180 K
0.20 × 0.20 × 0.15 mm

#### Data collection

Bruker–Nonius X8APEXII CCD diffractometer
Absorption correction: multi-scan (*SADABS*; Sheldrick, 2003 ▶) *T*_min_ = 0.701, *T*_max_ = 0.794
32580 measured reflections
4775 independent reflections
4248 reflections with *I* > 2σ(*I*)
*R*_int_ = 0.031

#### Refinement

*R*[*F* ^2^ > 2σ(*F* ^2^)] = 0.053
*wR*(*F* ^2^) = 0.132
*S* = 1.36
4775 reflections
199 parameters
H-atom parameters constrained
Δρ_max_ = 0.55 e Å^−3^
Δρ_min_ = −0.71 e Å^−3^

### 

Data collection: *APEX2* (Bruker, 2004 ▶); cell refinement: *SAINT* (Bruker, 2003 ▶); data reduction: *SAINT*; program(s) used to solve structure: *SHELXTL* (Sheldrick, 2008 ▶); program(s) used to refine structure: *SHELXTL*; molecular graphics: *SHELXTL*; software used to prepare material for publication: *SHELXTL*.

## Supplementary Material

Crystal structure: contains datablocks global, I. DOI: 10.1107/S1600536809051022/jh2118sup1.cif

Structure factors: contains datablocks I. DOI: 10.1107/S1600536809051022/jh2118Isup2.hkl

Additional supplementary materials:  crystallographic information; 3D view; checkCIF report

## Figures and Tables

**Table 1:** Hydrogen-bond geometry (Å, °)

*D*—H⋯*A*	*D*—H	H⋯*A*	*D*⋯*A*	*D*—H⋯*A*
O2—H2⋯O2^i^	0.93	1.64	2.559 (6)	169

Symmetry code: (i) 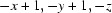.

**Fig. 1.**
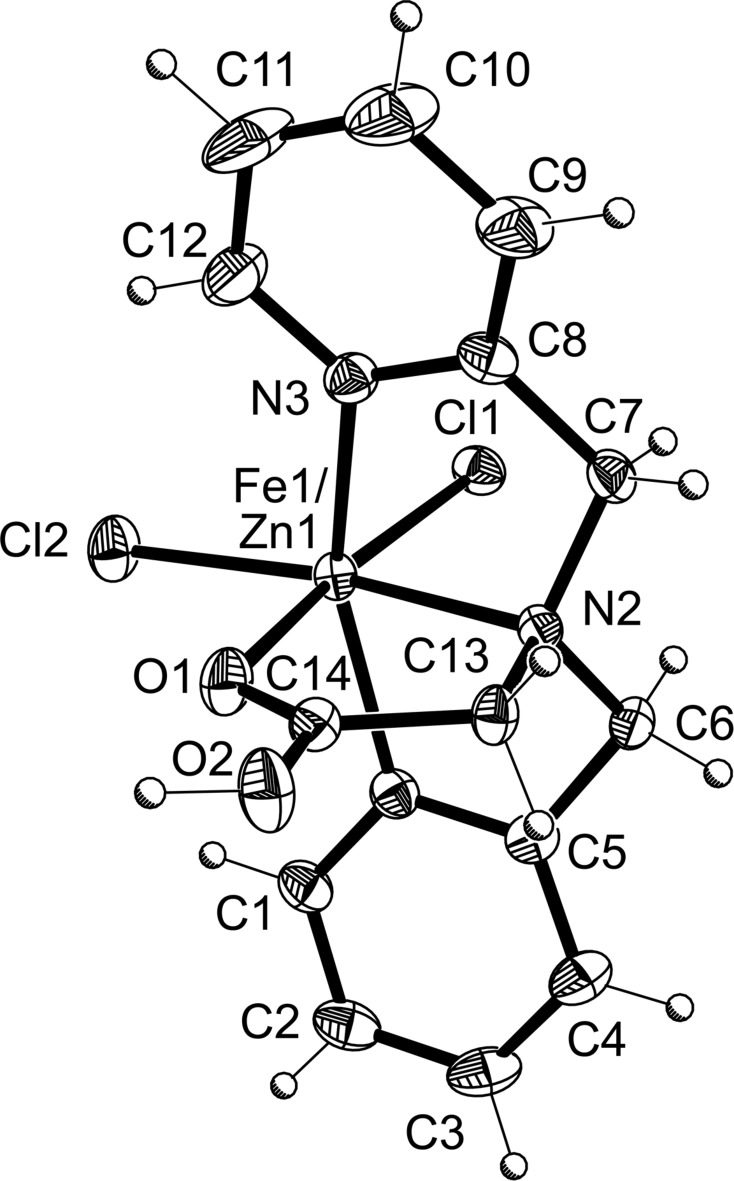
Molecular structure with displacement ellipsoids shown at 50% probability for non-H atoms.

**Fig. 2.**
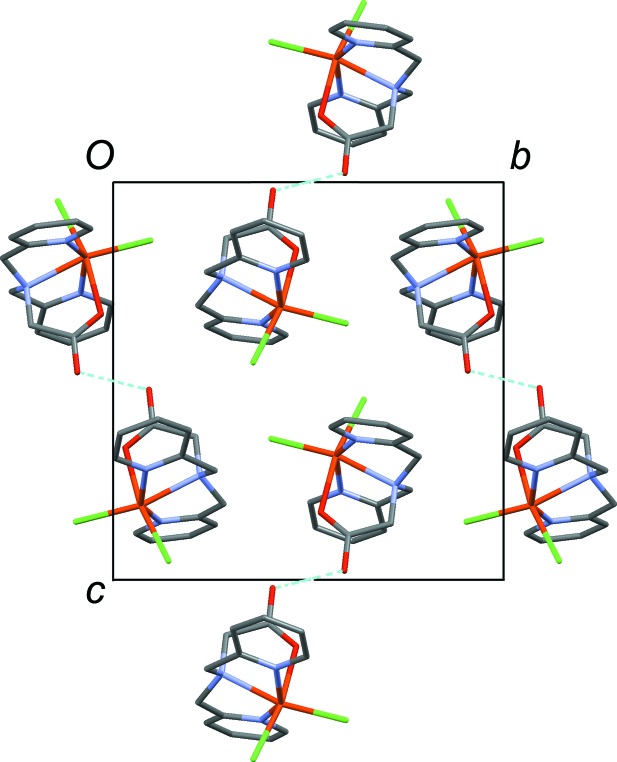
Projection along the a axis showing hydrogen bonds (dashed lines) formed between bpg(H) ligands across crystallographic inversion centres.

## supplementary crystallographic information

### Comment

Crystallization of the title compound, [FeCl_2_(bpg)][ZnCl_2_(bpgH)] (where bpg denotes the tetradentate ligand *N*,*N*-bis(2-pyridylmethyl)glycine), was surprising given the presence of water in its preparation. In our hands, simple binary mixtures of bpgH and FeCl_3_ in water-containing solutions do not yield the complex [FeCl_2_(bpg)]. If only chloride ions but not Zn^II^ are present in equivalent reaction mixtures (*i.e. *aerobic conditions and containing water), oligomeric (hydr)oxo-bridged Fe^III^ complexes are formed, such as [Fe_2_(O)(bpg)_2_(H_2_O)_2_](ClO_4_)_2_ and [Fe_3_(O)_2_(OH)(bpg)_3_](ClO_4_) (Mortensen *et al.*, 2004). Addition of Zn^II^ has in this case enabled isolation of [FeCl_2_(bpg)] from water as one component of the co-crystal.

### Experimental

*N*,*N*-Bis(2-pyridylmethyl)glycine (bpgH) was prepared according to a literature method (Suzuki *et al.*, 1988). BpgH (125 mg, 49 mmol) was then dissolved in hot acetonitrile (5 ml) and water (0.5 ml), before Fe(NO_3_)_3_.9H_2_O (99 mg, 24 mmol), ZnCl_2_ (67 mg, 49 mmol) and NH_4_Cl (78 mg, 15 mmol) were added. A few yellow crystals of the title compound were deposited overnight.

### Refinement

H atoms bound to C atoms were placed in idealized positions with C—H = 0.95 or 0.99 Å and refined as riding with *U*_iso_(H) = 1.2*U*_eq_(C). The H atom of the OH group was included in a position identified from a difference Fourier map, then allowed to ride on atom O2 with *U*_iso_(H) = 1.5*U*_eq_(O). The 50:50 disorder of atoms Fe1 and Zn1 is required for charge balance in the structure, but it is also supported by the diffraction data: refinement of the atom solely as Fe gives a comparatively small displacement ellipsoid while refinement solely as Zn gives a comparatively large ellipsoid. In both cases, the *R*-factors increase compared to the disordered refinement.

### Crystal data

**Table:**

[Fe(C_14_H_14_N_3_O_2_)Cl_2_]·[ZnCl_2_(C_14_H_15_N_3_O_2_)]	*F*(000) = 790
*M**_r_* = 776.59	*D*_x_ = 1.646 Mg m^−^^3^
Monoclinic, *P*2_1_/*c*	Mo *K*α radiation, λ = 0.71073 Å
Hall symbol: -P 2ybc	Cell parameters from 5945 reflections
*a* = 8.8710 (3) Å	θ = 2.3–29.9°
*b* = 13.1898 (5) Å	µ = 1.62 mm^−^^1^
*c* = 13.3983 (4) Å	*T* = 180 K
β = 91.737 (2)°	Block, yellow
*V* = 1566.97 (9) Å^3^	0.20 × 0.20 × 0.15 mm
*Z* = 2	

### Data collection

**Table:**

Bruker–Nonius X8APEXII CCD diffractometer	4775 independent reflections
Radiation source: fine-focus sealed tube	4248 reflections with *I* > 2σ(*I*)
graphite	*R*_int_ = 0.031
Thin–slice ω and φ scans	θ_max_ = 30.5°, θ_min_ = 3.8°
Absorption correction: multi-scan (*SADABS*; Sheldrick, 2003)	*h* = −12→12
*T*_min_ = 0.701, *T*_max_ = 0.794	*k* = −18→18
32580 measured reflections	*l* = −19→18

### Refinement

**Table:**

Refinement on *F*^2^	Primary atom site location: structure-invariant direct methods
Least-squares matrix: full	Secondary atom site location: difference Fourier map
*R*[*F*^2^ > 2σ(*F*^2^)] = 0.053	Hydrogen site location: inferred from neighbouring sites
*wR*(*F*^2^) = 0.132	H-atom parameters constrained
*S* = 1.36	*w* = 1/[σ^2^(*F*_o_^2^) + 7.0062*P*] where *P* = (*F*_o_^2^ + 2*F*_c_^2^)/3
4775 reflections	(Δ/σ)_max_ < 0.001
199 parameters	Δρ_max_ = 0.55 e Å^−^^3^
0 restraints	Δρ_min_ = −0.71 e Å^−^^3^

### Special details

**Table:**

Geometry. All e.s.d.'s (except the e.s.d. in the dihedral angle between two l.s. planes) are estimated using the full covariance matrix. The cell e.s.d.'s are taken into account individually in the estimation of e.s.d.'s in distances, angles and torsion angles; correlations between e.s.d.'s in cell parameters are only used when they are defined by crystal symmetry. An approximate (isotropic) treatment of cell e.s.d.'s is used for estimating e.s.d.'s involving l.s. planes.
Refinement. Refinement of *F*^2^ against ALL reflections. The weighted *R*-factor *wR* and goodness of fit *S* are based on *F*^2^, conventional *R*-factors *R* are based on *F*, with *F* set to zero for negative *F*^2^. The threshold expression of *F*^2^ > σ(*F*^2^) is used only for calculating *R*-factors(gt) *etc*. and is not relevant to the choice of reflections for refinement. *R*-factors based on *F*^2^ are statistically about twice as large as those based on *F*, and *R*- factors based on ALL data will be even larger.

### Fractional atomic coordinates and isotropic or equivalent isotropic displacement parameters (Å^2^)

**Table:**

	*x*	*y*	*z*	*U*_iso_*/*U*_eq_	Occ. (<1)
Fe1	0.30263 (5)	0.42993 (3)	0.31348 (3)	0.01709 (11)	0.50
Zn1	0.30263 (5)	0.42993 (3)	0.31348 (3)	0.01709 (11)	0.50
Cl1	0.19027 (10)	0.35846 (7)	0.45311 (7)	0.02319 (19)	
Cl2	0.30370 (13)	0.59544 (7)	0.35454 (8)	0.0290 (2)	
O1	0.3982 (4)	0.4689 (2)	0.1686 (2)	0.0295 (6)	
O2	0.4679 (4)	0.4082 (2)	0.0219 (2)	0.0326 (7)	
H2	0.4790	0.4759	0.0050	0.049*	0.50
N1	0.5237 (4)	0.3803 (2)	0.3539 (2)	0.0204 (6)	
N2	0.3235 (3)	0.2778 (2)	0.2375 (2)	0.0171 (6)	
N3	0.0989 (4)	0.4170 (3)	0.2225 (2)	0.0215 (6)	
C1	0.6376 (4)	0.4414 (3)	0.3838 (3)	0.0245 (8)	
H1A	0.6184	0.5117	0.3927	0.029*	
C2	0.7825 (5)	0.4048 (4)	0.4022 (3)	0.0291 (9)	
H2A	0.8617	0.4493	0.4228	0.035*	
C3	0.8095 (5)	0.3026 (4)	0.3899 (3)	0.0320 (10)	
H3A	0.9081	0.2759	0.4010	0.038*	
C4	0.6917 (5)	0.2392 (3)	0.3615 (3)	0.0280 (8)	
H4A	0.7076	0.1684	0.3548	0.034*	
C5	0.5506 (4)	0.2805 (3)	0.3429 (3)	0.0208 (7)	
C6	0.4167 (4)	0.2175 (3)	0.3096 (3)	0.0202 (7)	
H6A	0.4512	0.1543	0.2775	0.024*	
H6B	0.3564	0.1989	0.3678	0.024*	
C7	0.1677 (4)	0.2401 (3)	0.2253 (3)	0.0215 (7)	
H7A	0.1323	0.2150	0.2901	0.026*	
H7B	0.1647	0.1829	0.1774	0.026*	
C8	0.0656 (4)	0.3237 (3)	0.1875 (3)	0.0230 (7)	
C9	−0.0583 (5)	0.3063 (4)	0.1241 (3)	0.0340 (10)	
H9A	−0.0797	0.2399	0.1001	0.041*	
C10	−0.1499 (6)	0.3865 (5)	0.0964 (4)	0.0445 (13)	
H10A	−0.2355	0.3759	0.0534	0.053*	
C11	−0.1162 (6)	0.4821 (4)	0.1318 (4)	0.0465 (14)	
H11A	−0.1784	0.5382	0.1137	0.056*	
C12	0.0097 (5)	0.4952 (4)	0.1942 (4)	0.0336 (10)	
H12A	0.0338	0.5613	0.2178	0.040*	
C13	0.3965 (4)	0.2893 (3)	0.1407 (3)	0.0198 (7)	
H13A	0.3337	0.2553	0.0883	0.024*	
H13B	0.4956	0.2548	0.1442	0.024*	
C14	0.4195 (4)	0.3982 (3)	0.1113 (3)	0.0190 (7)	

### Atomic displacement parameters (Å^2^)

**Table:**

	*U*^11^	*U*^22^	*U*^33^	*U*^12^	*U*^13^	*U*^23^
Fe1	0.0191 (2)	0.0150 (2)	0.0172 (2)	−0.00111 (17)	0.00049 (15)	0.00011 (17)
Zn1	0.0191 (2)	0.0150 (2)	0.0172 (2)	−0.00111 (17)	0.00049 (15)	0.00011 (17)
Cl1	0.0217 (4)	0.0273 (5)	0.0207 (4)	−0.0020 (3)	0.0013 (3)	0.0032 (3)
Cl2	0.0400 (6)	0.0201 (4)	0.0272 (5)	0.0007 (4)	0.0063 (4)	−0.0067 (4)
O1	0.0351 (16)	0.0169 (13)	0.0363 (17)	0.0000 (12)	0.0006 (13)	−0.0069 (12)
O2	0.0476 (19)	0.0233 (15)	0.0271 (15)	−0.0051 (13)	0.0057 (13)	0.0043 (12)
N1	0.0204 (15)	0.0210 (15)	0.0196 (14)	−0.0020 (12)	−0.0012 (11)	−0.0008 (12)
N2	0.0173 (14)	0.0162 (14)	0.0178 (13)	−0.0016 (11)	−0.0002 (11)	0.0006 (11)
N3	0.0205 (15)	0.0223 (16)	0.0216 (15)	0.0013 (12)	−0.0015 (12)	−0.0004 (12)
C1	0.0231 (18)	0.0261 (19)	0.0243 (18)	−0.0055 (15)	0.0003 (14)	−0.0037 (15)
C2	0.0209 (18)	0.041 (2)	0.0253 (19)	−0.0060 (17)	−0.0018 (15)	−0.0064 (17)
C3	0.0211 (19)	0.047 (3)	0.028 (2)	0.0080 (18)	−0.0049 (15)	−0.0064 (19)
C4	0.026 (2)	0.031 (2)	0.0268 (19)	0.0081 (16)	−0.0041 (15)	−0.0039 (16)
C5	0.0213 (17)	0.0243 (18)	0.0166 (15)	0.0013 (14)	−0.0013 (13)	−0.0016 (13)
C6	0.0226 (17)	0.0161 (16)	0.0218 (17)	0.0015 (13)	−0.0010 (13)	0.0005 (13)
C7	0.0208 (17)	0.0186 (17)	0.0249 (17)	−0.0041 (14)	−0.0034 (13)	−0.0010 (14)
C8	0.0193 (17)	0.0273 (19)	0.0223 (17)	−0.0033 (15)	−0.0014 (13)	−0.0025 (15)
C9	0.027 (2)	0.040 (3)	0.034 (2)	−0.0024 (18)	−0.0101 (17)	−0.0074 (19)
C10	0.030 (2)	0.057 (3)	0.045 (3)	0.010 (2)	−0.018 (2)	−0.011 (2)
C11	0.036 (3)	0.051 (3)	0.052 (3)	0.022 (2)	−0.020 (2)	−0.011 (2)
C12	0.031 (2)	0.029 (2)	0.041 (3)	0.0087 (18)	−0.0066 (18)	−0.0021 (19)
C13	0.0267 (18)	0.0161 (16)	0.0168 (15)	0.0002 (13)	0.0028 (13)	0.0007 (12)
C14	0.0204 (16)	0.0185 (16)	0.0179 (16)	0.0000 (13)	−0.0005 (13)	−0.0019 (13)

### Geometric parameters (Å, °)

**Table:**

Fe1—N1	2.122 (3)	C3—H3A	0.950
Fe1—N3	2.156 (3)	C4—C5	1.381 (5)
Fe1—O1	2.202 (3)	C4—H4A	0.950
Fe1—Cl2	2.2512 (11)	C5—C6	1.506 (5)
Fe1—N2	2.260 (3)	C6—H6A	0.990
Fe1—Cl1	2.3441 (10)	C6—H6B	0.990
O1—C14	1.226 (5)	C7—C8	1.505 (5)
O2—C14	1.291 (5)	C7—H7A	0.990
O2—H2	0.927	C7—H7B	0.990
N1—C1	1.345 (5)	C8—C9	1.387 (5)
N1—C5	1.346 (5)	C9—C10	1.378 (7)
N2—C7	1.473 (5)	C9—H9A	0.950
N2—C13	1.475 (5)	C10—C11	1.377 (8)
N2—C6	1.483 (5)	C10—H10A	0.950
N3—C8	1.347 (5)	C11—C12	1.386 (6)
N3—C12	1.348 (5)	C11—H11A	0.950
C1—C2	1.388 (6)	C12—H12A	0.950
C1—H1A	0.950	C13—C14	1.504 (5)
C2—C3	1.381 (7)	C13—H13A	0.990
C2—H2A	0.950	C13—H13B	0.990
C3—C4	1.382 (6)		
			
N1—Fe1—N3	150.70 (12)	C3—C4—H4A	120.5
N1—Fe1—O1	85.37 (12)	N1—C5—C4	121.9 (4)
N3—Fe1—O1	81.87 (12)	N1—C5—C6	115.5 (3)
N1—Fe1—Cl2	103.88 (9)	C4—C5—C6	122.6 (4)
N3—Fe1—Cl2	102.26 (9)	N2—C6—C5	108.4 (3)
O1—Fe1—Cl2	89.45 (8)	N2—C6—H6A	110.0
N1—Fe1—N2	75.71 (11)	C5—C6—H6A	110.0
N3—Fe1—N2	75.74 (12)	N2—C6—H6B	110.0
O1—Fe1—N2	76.77 (11)	C5—C6—H6B	110.0
Cl2—Fe1—N2	166.22 (8)	H6A—C6—H6B	108.4
N1—Fe1—Cl1	94.84 (9)	N2—C7—C8	110.1 (3)
N3—Fe1—Cl1	92.86 (9)	N2—C7—H7A	109.6
O1—Fe1—Cl1	168.98 (8)	C8—C7—H7A	109.6
Cl2—Fe1—Cl1	101.17 (4)	N2—C7—H7B	109.6
N2—Fe1—Cl1	92.57 (8)	C8—C7—H7B	109.6
C14—O1—Fe1	116.5 (3)	H7A—C7—H7B	108.2
C14—O2—H2	111.5	N3—C8—C9	121.7 (4)
C1—N1—C5	119.0 (3)	N3—C8—C7	115.4 (3)
C1—N1—Fe1	124.9 (3)	C9—C8—C7	122.8 (4)
C5—N1—Fe1	116.1 (2)	C10—C9—C8	119.2 (4)
C7—N2—C13	111.8 (3)	C10—C9—H9A	120.4
C7—N2—C6	113.2 (3)	C8—C9—H9A	120.4
C13—N2—C6	112.1 (3)	C11—C10—C9	119.4 (4)
C7—N2—Fe1	105.1 (2)	C11—C10—H10A	120.3
C13—N2—Fe1	110.4 (2)	C9—C10—H10A	120.3
C6—N2—Fe1	103.6 (2)	C10—C11—C12	119.0 (5)
C8—N3—C12	118.7 (4)	C10—C11—H11A	120.5
C8—N3—Fe1	116.2 (3)	C12—C11—H11A	120.5
C12—N3—Fe1	125.0 (3)	N3—C12—C11	122.0 (5)
N1—C1—C2	121.9 (4)	N3—C12—H12A	119.0
N1—C1—H1A	119.1	C11—C12—H12A	119.0
C2—C1—H1A	119.1	N2—C13—C14	113.3 (3)
C3—C2—C1	118.8 (4)	N2—C13—H13A	108.9
C3—C2—H2A	120.6	C14—C13—H13A	108.9
C1—C2—H2A	120.6	N2—C13—H13B	108.9
C2—C3—C4	119.4 (4)	C14—C13—H13B	108.9
C2—C3—H3A	120.3	H13A—C13—H13B	107.7
C4—C3—H3A	120.3	O1—C14—O2	124.4 (4)
C5—C4—C3	119.0 (4)	O1—C14—C13	122.5 (3)
C5—C4—H4A	120.5	O2—C14—C13	113.1 (3)
			
N1—Fe1—O1—C14	77.2 (3)	C5—N1—C1—C2	1.1 (6)
N3—Fe1—O1—C14	−76.3 (3)	Fe1—N1—C1—C2	−175.2 (3)
Cl2—Fe1—O1—C14	−178.8 (3)	N1—C1—C2—C3	−0.5 (6)
N2—Fe1—O1—C14	0.8 (3)	C1—C2—C3—C4	−1.0 (7)
Cl1—Fe1—O1—C14	−14.3 (7)	C2—C3—C4—C5	2.0 (6)
N3—Fe1—N1—C1	144.7 (3)	C1—N1—C5—C4	−0.1 (6)
O1—Fe1—N1—C1	80.4 (3)	Fe1—N1—C5—C4	176.5 (3)
Cl2—Fe1—N1—C1	−7.9 (3)	C1—N1—C5—C6	179.8 (3)
N2—Fe1—N1—C1	157.9 (3)	Fe1—N1—C5—C6	−3.7 (4)
Cl1—Fe1—N1—C1	−110.7 (3)	C3—C4—C5—N1	−1.5 (6)
N3—Fe1—N1—C5	−31.7 (4)	C3—C4—C5—C6	178.7 (4)
O1—Fe1—N1—C5	−96.0 (3)	C7—N2—C6—C5	−160.8 (3)
Cl2—Fe1—N1—C5	175.7 (3)	C13—N2—C6—C5	71.5 (4)
N2—Fe1—N1—C5	−18.5 (3)	Fe1—N2—C6—C5	−47.5 (3)
Cl1—Fe1—N1—C5	73.0 (3)	N1—C5—C6—N2	36.7 (4)
N1—Fe1—N2—C7	154.6 (2)	C4—C5—C6—N2	−143.5 (4)
N3—Fe1—N2—C7	−32.0 (2)	C13—N2—C7—C8	−75.2 (4)
O1—Fe1—N2—C7	−116.9 (2)	C6—N2—C7—C8	157.0 (3)
Cl2—Fe1—N2—C7	−115.3 (4)	Fe1—N2—C7—C8	44.6 (3)
Cl1—Fe1—N2—C7	60.3 (2)	C12—N3—C8—C9	−0.5 (6)
N1—Fe1—N2—C13	−84.6 (2)	Fe1—N3—C8—C9	−176.8 (3)
N3—Fe1—N2—C13	88.7 (2)	C12—N3—C8—C7	−178.1 (4)
O1—Fe1—N2—C13	3.9 (2)	Fe1—N3—C8—C7	5.6 (4)
Cl2—Fe1—N2—C13	5.4 (5)	N2—C7—C8—N3	−35.6 (5)
Cl1—Fe1—N2—C13	−179.0 (2)	N2—C7—C8—C9	146.9 (4)
N1—Fe1—N2—C6	35.6 (2)	N3—C8—C9—C10	−0.2 (7)
N3—Fe1—N2—C6	−151.1 (2)	C7—C8—C9—C10	177.2 (4)
O1—Fe1—N2—C6	124.1 (2)	C8—C9—C10—C11	0.3 (8)
Cl2—Fe1—N2—C6	125.6 (3)	C9—C10—C11—C12	0.2 (9)
Cl1—Fe1—N2—C6	−58.8 (2)	C8—N3—C12—C11	1.1 (7)
N1—Fe1—N3—C8	28.4 (4)	Fe1—N3—C12—C11	177.0 (4)
O1—Fe1—N3—C8	93.5 (3)	C10—C11—C12—N3	−1.0 (9)
Cl2—Fe1—N3—C8	−178.8 (3)	C7—N2—C13—C14	109.1 (3)
N2—Fe1—N3—C8	15.2 (3)	C6—N2—C13—C14	−122.4 (3)
Cl1—Fe1—N3—C8	−76.7 (3)	Fe1—N2—C13—C14	−7.5 (4)
N1—Fe1—N3—C12	−147.6 (3)	Fe1—O1—C14—O2	176.6 (3)
O1—Fe1—N3—C12	−82.5 (4)	Fe1—O1—C14—C13	−5.8 (5)
Cl2—Fe1—N3—C12	5.2 (4)	N2—C13—C14—O1	9.3 (5)
N2—Fe1—N3—C12	−160.8 (4)	N2—C13—C14—O2	−172.8 (3)
Cl1—Fe1—N3—C12	107.3 (4)		

### Hydrogen-bond geometry (Å, °)

**Table:**

*D*—H···*A*	*D*—H	H···*A*	*D*···*A*	*D*—H···*A*
O2—H2···O2^i^	0.93	1.64	2.559 (6)	169

Symmetry codes: (i) −*x*+1, −*y*+1, −*z*.
